# Further results on robust stability for uncertain neutral systems with distributed delay

**DOI:** 10.1186/s13660-018-1911-8

**Published:** 2018-11-16

**Authors:** Tao Wu, Lianglin Xiong, Jinde Cao, Xinzhi Liu

**Affiliations:** 1 School of Mathematics and Computer Science, Yunnan Minzu University, Kunming, China; 2 School of Mathematics, Southeast University, Nanjing, China; 3 Department of Applied Mathematics, University of Waterloo, Waterloo, Canada

**Keywords:** Robust stability, Uncertain neutral delayed system, Delay-dependent stability, Lyapunov functional, Linear matrix inequalities

## Abstract

This paper studies the problem of robust stability for uncertain neutral systems with distributed delay. By utilizing the incorporation of a new integral inequality technique and a novel Lyapunov–Krasovskii functional, some reduced conservative delay-dependent stability conditions for asymptotic stability are established. Then some special cases of neutral systems are discussed. Based on these delay-dependent stability conditions, the condition for robustness is obtained for uncertain linear delayed systems. All these stability conditions are given in terms of linear matrix inequalities (LMIs), which can easily be computed by the LMI toolbox of Matlab. Finally, several examples are discussed in detail to display the usefulness and superiority of the obtained results.

## Introduction

The stability analysis of neutral delay-differential systems has received considerable attention over the decades [1–18]. In the literature [1–18], the W-transform approach [1], the positivity-based approach [2–16], the characteristic equation method [17], the Lyapunov technique [18], and the state trajectory approach have been utilized to derive sufficient conditions for asymptotic stability and exponential stability of the systems. However, most of the criteria are expressed in terms of a matrix norm or matrix measure of the system matrices. Unfortunately, the matrix norm operations usually make the criteria more conservative. Also the criteria in recent studies [16–18] require strong assumptions such as that the matrix measures of system matrices have to be negative. These assumptions often make it difficult to apply the criteria to various systems, such as neutral delay-differential systems with time-varying delay, uncertain neutral delay-differential systems with time-delay, and so on. Inversely, these problems can easily be solved via the Lyapunov–Krasovskii functional (LKF) method, and this method has thus received considerable attention in the area of control engineering (see [19–23]).

As is well known, the Lyapunov functional method has received more and more attention in recent years due to its effectiveness in many problems, such as the problem of control for linear neutral systems, the problem of delay-dependent stability (DDS) for linear neutral systems (LNSS), etc. Based on this method, many interesting results on less conservative DDS conditions have been obtained (see, e.g., [24–42]). It is worth noting that [24–30, 42] mainly considered the neutral and discrete delay. In fact, a lot of practical applications are modeled by systems with distributed delay. Consequently, a large number of stability and stabilization results on systems with distributed delay have been reported in [31–40]. It is pointed out that, although the information of three kinds of time-delays were considered in [32–37], the relationships between the three kinds of time-delays have not been fully studied. Accordingly, their interrelationships are discussed in [41]. However, so far, the obtained maximum allowable upper bounds (MAUBs) of delay in [41] are not the best results. Thus, there still exists some room to improve with some novel LKFs, which combines with a new inequality technique.

On the other hand, DDS conditions are often obtained by the Lyapunov functionals theorem accompanied by some important techniques. Many important methods include the bounding inequalities for the cross term [24], a descriptor model transformation [25], the free-weighting matrix technique [26], integral inequality (II) methods (see [27, 28, 42]), delay decomposition [29], and discretized LKFs [30]. Generally speaking, the II method has always played a very important role in acquiring a DDS condition.

Up to now, many outstanding efforts have been made focused on the investigation of integral inequalities to decrease the conservatism of stability criteria. As an option of the Jensen inequality [25], the Wirtinger-based inequality was presented [43], in which the single integral information of the state is considered. Recently, the free-matrix based II was proposed in [44] by introducing some free matrices, which may be considered as an improved version of the Wirtinger-based inequality. More recently, the double integral information and triple integral information of the states was considered and a proposal was made [45] which could lead to more relaxed stability criteria for the systems. Specially, it is noted that in order to further reduce the conservatism, some triple integral functions have been added into the LKFs [45]. Very recently, [46] presented a series of single/multiple IIs which proved to be less conservative than the existing ones. With the results in [46], further less conservative results would be obtained based on some novel Lyapunov functionals. Hence, the IIs in [46] should not be ignored, because they maybe play a significant role in getting a delay-dependent stability condition for neutral systems with discrete and distributed delays.

Motivated by the above discussion, the uncertain LNSS with discrete and distributed delays will be considered in this article. By choosing some suitable IIs, a novel LKF is constructed. Based on the Lyapunov stability theory, some DDS conditions are got in terms of LMIs. Moreover, some examples are given to display the superiority and low conservatism of our results.

Some significant symbols used throughout this paper are considerably standard. The symbols ‘−1’ and ‘*T*’ represent the inverse and transpose of a matrix, respectively; $\mathbb{R}^{n}$ stands for n-dimensional Euclidean space; $\mathbb{R}^{m \times n}$ is the set of all $m\times n$ real matrices; $P>0$ means that the matrix *P* is symmetric and positive definite; $\operatorname{sym} \{{X} \} =X+X^{T}$; *I* is the identity matrix; 0 is a zero matrix; and $\Vert \cdot \Vert $ refers to the induced matrix 2-norm. If the dimensions of a matrix are not explicitly stated, the matrix is assumed to have compatible dimensions.

## Problem statement

Consider the uncertain LNSS with time-delay

1 $$ \textstyle\begin{cases} \dot{x}(t) - \mathbf{B}\dot{x}(t - \tau)= \mathbf{A}x(t)+{\mathbf {A}_{1}}x(t - h)+ {\mathbf{A}_{2}}\int_{t - r}^{t} {x(s)} \,ds, \\ x(t)= \phi(t),\quad \forall t \in [ { - \rho,0} ],\rho = \max \{ {\tau,h,r} \},t \ge0, \end{cases} $$

where $x(t)\in\mathbb{R}^{n}$ is the state vector, *τ*, *h* and *r* represent the neutral, discrete and distributed delay, respectively. $\rho= \max\{\tau, h,r\}$, $\phi ( t )$ is the initial condition function. $\mathbf{B}=B + \Delta B$, $\mathbf{A}=A + \Delta A$, ${\mathbf{A}_{1}}={A_{1}}+ \Delta{A_{1}}$, ${\mathbf{A}_{2}}={A_{2}} + \Delta {A_{2}}$. *A*, *B*, ${A_{1}}$, ${A_{2}}$ are known constant matrices. Δ*A*, $\Delta{A_{1}}$, $\Delta{A_{2}}$ and Δ*B* denote the time-varying uncertainties, and the uncertainties are supposed to be norm-bounded and satisfy

2 $$\left[\begin{array}{cccc} \Delta A & \Delta A_{1} & \Delta A_{2} & \Delta B \end{array}\right]=DF(t)\left[\begin{array}{cccc} N_{a} & N_{b} & N_{c} & N_{d} \end{array}\right],$$

where *D*, ${N_{a}}$, ${N_{b}}$, ${N_{c}}$ and ${N_{d}}$ are known constant matrices, and $F(t)$ is an unknown continuous time-varying matrix function and satisfies ${F^{T}}(t)F(t) \le I$. Furthermore, for system (1), in order to guarantee system (1) has asymptotic stability, we need to assume $\Vert {B + \Delta B} \Vert \le1$.

The major object of this paper is to establish some less conservative stability and robust stability conditions (RSCSs) for system (1). Before giving the primary results of this article, some important lemmas are firstly introduced as follows.

### Lemma 2.1

([45])

*For a given* $R>0$ *and any differentiable function* $w: [ {a,b} ] \to{\mathbb{R}^{n}}$, *the following inequality holds*:

3 $$\begin{aligned}& \int_{a}^{b} {{w^{T}}(\alpha)Rw(\alpha)\,d \alpha} \ge\frac{1}{{b - a}}{ \biggl( { \int_{a}^{b} {w(\alpha)\,d\alpha} } \biggr)^{T}}R \biggl( { \int_{a}^{b} {w(\alpha)\,d\alpha} } \biggr), \end{aligned}$$

4 $$\begin{aligned}& \begin{aligned}[b] \int_{a}^{b} {{w^{T}}(\alpha)Rw(\alpha)\,d \alpha}&\ge \frac{1}{{b - a}}{ \biggl( { \int_{a}^{b} {w(\alpha)\,d\alpha} } \biggr)^{T}}R \biggl( { \int_{a}^{b} {w(\alpha)\,d\alpha} } \biggr) \\ &\quad {}+ \frac{3}{{b - a}}{\bar{\varOmega}_{1}}^{T}R{\bar{\varOmega}_{1}} + \frac{5}{{b - a}}{\bar{\varOmega}_{2}}^{T}R{ \bar{\varOmega}_{2}}, \end{aligned} \end{aligned}$$

*where*

$$\begin{aligned}& {\bar{\varOmega}_{1}} = \int_{a}^{b} {w(\alpha)\,d\alpha} - \frac{2}{{b - a}} \int_{a}^{b} { \int_{\beta}^{b} {w(\alpha)\,d\alpha \,d\beta} }, \\& {\bar{\varOmega}_{2}} = \int_{a}^{b} {w(\alpha)\,d\alpha} - \frac{6}{{b - a}} \int_{a}^{b} { \int_{\beta}^{b} {w(\alpha)\,d\alpha \,d\beta} }+ \frac {{12}}{{{{ ( {b - a} )}^{2}}}} \int_{a}^{b} { \int_{\gamma}^{b} { \int _{\beta}^{b} {w(\alpha)\,d\alpha \,d\beta \,d \gamma} } }. \end{aligned}$$

### Lemma 2.2

([46])

*For a given* $R>0$ *and any differentiable function* $x: [ {a,b} ] \to{\mathbb{R}^{n}}$, *and* ${M_{k}}\in{\mathbb{R}^{6n \times n}}$ ($k = 1,2,3,4,5$), *the following inequality holds*:

5 $$ - \int_{a}^{b} {{{\dot{x}}^{T}}(s)R\dot{x}(s) \,ds} \le{\upsilon^{T}}\varOmega \upsilon, $$

*where*

$$\begin{array}{rl} & \upsilon ={\left[\begin{array}{cccccc} x^{T}(b) & x^{T}(a) & \frac{1}{b_{a}}v_{1}^{T} & \frac{2}{b_{a}^{2}}v_{2}^{T} & \frac{6}{b_{a}^{3}}v_{3}^{T} & \frac{24}{b_{a}^{4}}v_{4}^{T} \end{array}\right]}^{T},\\ & \Omega =\sum_{k=1}^{5}\frac{b_{a}}{2k-1}M_{k}R^{-1}M_{k}^{T}+\mathrm{sym}\{M_{k}\Pi_{k}\},\;b_{a}=b-a,\\ & v_{1}=\int_{a}^{b}x(s)\;ds,\;v_{2}=\int_{a}^{b}\int_{\theta }^{b}x(s)\;ds\;d\theta ,\;v_{3}=\int_{a}^{b}\int_{\theta }^{b}\int_{u}^{b}x(s)\;ds\;du\;d\theta ,\\ & v_{4}=\int_{a}^{b}\int_{\theta }^{b}\int_{u}^{b}\int_{v}^{b}x(s)\;ds\;dv\;du\;d\theta ,\;\Pi_{1}={\bar{e}}_{1}-{\bar{e}}_{2},\;\Pi_{2}={\bar{e}}_{1}+{\bar{e}}_{2}-2{\bar{e}}_{3},\\ & \Pi_{3}={\bar{e}}_{1}-{\bar{e}}_{2}+6{\bar{e}}_{3}-6{\bar{e}}_{4},\;\Pi_{4}={\bar{e}}_{1}+{\bar{e}}_{2}-12{\bar{e}}_{3}+30{\bar{e}}_{4}-20{\bar{e}}_{5},\\ & \Pi_{5}={\bar{e}}_{1}-{\bar{e}}_{2}+20{\bar{e}}_{3}-90{\bar{e}}_{4}+140{\bar{e}}_{5}-70{\bar{e}}_{6},\\ & {\bar{e}}_{i}=\left[\begin{array}{ccc} 0_{n\times (i-1)n} & I_{n} & 0_{n\times (6-i)n} \end{array}\right],\;i=1,2,\ldots ,6. \end{array}$$

In the following, we will use Lemmas 2.1 and 2.2 to obtain some main results.

## Main results

In this section, for the uncertain LNSS (1), we give some sufficient conditions to ensure its robust stability. Then, for the sake of convenient calculation, we rewrite system (1) as

6 $$ \textstyle\begin{cases} \dot{x}(t)= Ax(t) + {A_{1}}x(t - h) + {A_{2}}\int_{t - r}^{t} {x(s)}\,ds+ B\dot{x}(t - \tau)+ Dp(t), \\ p(t)= F(t)q(t), \\ q(t)= {N_{a}}x(t) + {N_{b}}x(t - h) + {N_{c}}\int_{t - r}^{t} {x(s)} \,ds+ {N_{d}}\dot{x}(t - \tau), \end{cases} $$

where $p(t)\in{\mathbb{R}^{n}}$, $q(t)\in{\mathbb{R}^{n}}$. Now, the DDS conditions are presented by taking advantage of the new inequality technique and the novel constructed Lyapunov functionals.

### Theorem 3.1

*For given delays* *τ*, *h* *and* *r*, *system* (1) *is robustly asymptotically stable*, *if there exist positive definite matrices* $P \in\mathbb{R}^{5n \times5n}$, *and* $H, Q_{i}, R_{i} \in\mathbb{R}^{n\times n}$ ($i \in{1,2,3}$), $Q_{4} \in\mathbb {R}^{2n\times2n}$ *and any matrices* $N_{k}, M_{k}, F_{k}\in\mathbb {R}^{5n\times n}$ ($k \in{1,2,3,4}$) *satisfying the following inequalities*:

7 $$\begin{aligned} \varPsi&= \operatorname{sym} \bigl\{ {\varGamma_{2}^{T}P{ \varGamma_{1}}} \bigr\} + e_{1}^{T}({Q_{1}}+{Q_{2}}+r{Q_{3}}){e_{1}} - e_{2}^{T}{Q_{1}} {e_{2}}- e_{3}^{T}{Q_{2}} {e_{3}}- re_{12}^{T}{Q_{3}} {e_{12}} \\ &\quad {}-3r\varOmega_{1}^{T}{Q_{3}} { \varOmega_{1}}- 5r\varOmega_{2}^{T}{Q_{3}} {\varOmega_{2}}+ \varTheta _{1}^{T}{Q_{4}} {\varTheta_{1}} - \varTheta_{2}^{T}{Q_{4}} {\varTheta_{2}} + he_{0}^{T}{R_{1}} {e_{0}} \\ &\quad {} + \tau e_{0}^{T}{R_{2}} {e_{0}} + re_{0}^{T}{R_{3}} {e_{0}}+{\varOmega_{3}}+{\varOmega_{4}}+ { \varOmega_{5}} + {\varSigma^{T}}H\varSigma - e_{15}^{T}H{e_{15}}< 0, \end{aligned}$$

*where*

$$\begin{array}{rl} & \Gamma_{1}={\left[\begin{array}{ccccc} e_{1}^{T} & e_{3}^{T} & he_{6}^{T} & \tau e_{9}^{T} & re_{12}^{T} \end{array}\right]}^{T},\\ & \Gamma_{2}={\left[\begin{array}{ccccc} e_{0}^{T} & e_{5}^{T} & e_{1}^{T}-e_{2}^{T} & e_{1}^{T}-e_{3}^{T} & e_{1}^{T}-e_{4}^{T} \end{array}\right]}^{T},\\ & \Omega_{1}=e_{12}-2e_{13},\;\Omega_{2}=e_{12}-6e_{13}+12e_{14},\;\Theta_{1}={\left[\begin{array}{cc} e_{1}^{T} & e_{0}^{T} \end{array}\right]}^{T},\\ & \Theta_{2}={\left[\begin{array}{cc} e_{3}^{T} & e_{5}^{T} \end{array}\right]}^{T},\;e_{0}=Ae_{1}+A_{1}e_{2}+rA_{2}e_{12}+Be_{5}+De_{15},\\ & \upsilon_{1}={\left[\begin{array}{ccccc} e_{1}^{T} & e_{2}^{T} & e_{6}^{T} & e_{7}^{T} & e_{8}^{T} \end{array}\right]}^{T},\;\upsilon_{2}={\left[\begin{array}{ccccc} e_{1}^{T} & e_{2}^{T} & e_{9}^{T} & e_{10}^{T} & e_{11}^{T} \end{array}\right]}^{T},\\ & \upsilon_{3}={\left[\begin{array}{ccccc} e_{1}^{T} & e_{2}^{T} & e_{12}^{T} & e_{13}^{T} & e_{14}^{T} \end{array}\right]}^{T},\;\Sigma =N_{a}e_{1}+N_{b}e_{2}+rN_{c}e_{12}+N_{d}e_{5},\\ & \Omega_{3}=\upsilon_{1}^{T}\left(\sum_{k=1}^{4}\frac{h}{2k-1}N_{k}R_{1}^{-1}N_{k}^{T}\right)\upsilon_{1}+\mathrm{sym}\left\{\upsilon_{1}^{T}N_{k}E_{k}\right\},\\ & \Omega_{4}=\upsilon_{2}^{T}\left(\sum_{k=1}^{4}\frac{\tau }{2k-1}M_{k}R_{2}^{-1}M_{k}^{T}\right)\upsilon_{2}+\mathrm{sym}\left\{\upsilon_{2}^{T}M_{k}D_{k}\right\},\\ & \Omega_{5}=\upsilon_{3}^{T}\left(\sum_{k=1}^{4}\frac{r}{2k-1}F_{k}R_{3}^{-1}F_{k}^{T}\right)\upsilon_{3}+\mathrm{sym}\left\{\upsilon_{3}^{T}F_{k}G_{k}\right\},\\ & E_{1}=e_{1}-e_{2},\;E_{2}=e_{1}+e_{2}-2e_{6},\;E_{3}=e_{1}-e_{2}+6e_{6}-6e_{7},\\ & E_{4}=e_{1}+e_{2}-12e_{6}+30e_{7}-20e_{8},\\ & D_{1}=e_{1}-e_{3},\;D_{2}=e_{1}+e_{3}-2e_{9},\;D_{3}=e_{1}-e_{3}+6e_{9}-6e_{10},\\ & D_{4}=e_{1}+e_{3}-12e_{9}+30e_{10}-20e_{11},\\ & G_{1}=e_{1}-e_{4},\;G_{2}=e_{1}+e_{4}-2e_{12},\;G_{3}=e_{1}-e_{4}+6e_{12}-6e_{13},\\ & G_{4}=e_{1}+e_{4}-12e_{12}+30e_{13}-20e_{14},\\ & e_{i}=\left[\begin{array}{ccc} 0_{n\times (i-1)n} & I_{n} & 0_{n\times (15-i)n} \end{array}\right],\;i=1,2,3,\ldots ,15. \end{array}$$

### Proof

Take the following LKF candidate:

8 $$\begin{array}{rl} V(t) & =\eta_{1}^{T}(t)P\eta_{1}(t)+\int_{t-h}^{t}x^{T}(s)Q_{1}x(s)\;ds+\int_{t-\tau }^{t}x^{T}(s)Q_{2}x(s)\;ds\\ & \;+\int_{t-r}^{t}\int_{\theta }^{t}x^{T}(s)Q_{3}x(s)\;ds\;d\theta +\int_{t-\tau }^{t}{\left(\begin{array}{c} x(s)\\ \dot{x}(s) \end{array}\right)}^{T}Q_{4}\left(\begin{array}{c} x(s)\\ \dot{x}(s) \end{array}\right)\;ds\\ & \;+\int_{t-h}^{t}\int_{\theta }^{t}{\dot{x}}^{T}(s)R_{1}\dot{x}(s)\;ds\;d\theta +\int_{t-\tau }^{t}\int_{\theta }^{t}{\dot{x}}^{T}(s)R_{2}\dot{x}(s)\;ds\;d\theta \\ & \;+\int_{t-r}^{t}\int_{\theta }^{t}{\dot{x}}^{T}(s)R_{3}\dot{x}(s)\;ds\;d\theta , \end{array}$$

where

$$\eta_{1}(t)={\left[\begin{array}{ccccc} x^{T}(t) & x^{T}(t-\tau ) & \int_{t-h}^{t}x^{T}(s)\;ds & \int_{t-\tau }^{t}x^{T}(s)\;ds & \int_{t-r}^{t}x^{T}(s)\;ds \end{array}\right]}^{T}.$$

Define

9 $$\begin{array}{rl} \xi (t) & =[\begin{array}{ccccccc} x^{T}(t) & x^{T}(t-h) & x^{T}(t-\tau ) & x^{T}(t-r) & {\dot{x}}^{T}(t-\tau ) & \frac{1}{h}c_{1}^{T} & \frac{2}{h^{2}}c_{2}^{T} \end{array}\\ & \;{\begin{array}{cccccccc} \frac{6}{h^{3}}c_{3}^{T} & \frac{1}{\tau }c_{4}^{T} & \frac{2}{\tau^{2}}c_{5}^{T} & \frac{6}{\tau^{3}}c_{6}^{T} & \frac{1}{r}c_{7}^{T} & \frac{2}{r^{2}}c_{8}^{T} & \frac{6}{r^{3}}c_{9}^{T} & p^{T}(t) \end{array}]}^{T}, \end{array}$$

with

$$\begin{aligned}& {c_{1}} = \int_{t - h}^{t} {x ( s )} \,ds,\qquad {c_{2}} = \int_{t - h}^{t} { \int_{\theta}^{t} {x ( s )} } \,ds\,d\theta,\qquad {c_{3}} = \int_{t - h}^{t} { \int_{\theta}^{t} { \int_{u}^{t} {x ( s )} } } \,ds\,du\,d\theta, \\& {c_{4}} = \int_{t - \tau}^{t} {x ( s )} \,ds,\qquad {c_{5}} = \int_{t - \tau}^{t} { \int_{\theta}^{t} {x ( s )} } \,ds\,d\theta,\qquad {c_{6}} = \int_{t - \tau}^{t} { \int_{\theta}^{t} { \int_{u}^{t} {x ( s )} } } \,ds\,du\,d\theta, \\& {c_{7}} = \int_{t - r}^{t} {x ( s )} \,ds,\qquad {c_{8}} = \int_{t - r}^{t} { \int_{\theta}^{t} {x ( s )} } \,ds\,d\theta,\qquad {c_{9}} = \int_{t - r}^{t} { \int_{\theta}^{t} { \int_{u}^{t} {x ( s )} } } \,ds\,du\,d\theta. \end{aligned}$$

From (8), differentiating $V(t)$ leads to

10 $$\begin{array}{rl} \dot{V}(t) & =2{\dot{\eta }}_{1}^{T}(t)P\eta_{1}(t)+x^{T}(t)Q_{1}x(t)+x^{T}(t)Q_{2}x(t)-x^{T}(t-h)Q_{1}x(t-h)\\ & \;+h{\dot{x}}^{T}(t)R_{1}\dot{x}(t)-x^{T}(t-\tau )Q_{2}x(t-\tau )+rx^{T}(t)Q_{3}x(t)\\ & \;-\int_{t-r}^{t}x^{T}(s)Q_{3}x(s)\;ds+{\left(\begin{array}{c} x(t)\\ \dot{x}(t) \end{array}\right)}^{T}Q_{4}\left(\begin{array}{c} x(t)\\ \dot{x}(t) \end{array}\right)\\ & \;-{\left(\begin{array}{c} x(t-\tau )\\ \dot{x}(t-\tau ) \end{array}\right)}^{T}Q_{4}\left(\begin{array}{c} x(t-\tau )\\ \dot{x}(t-\tau ) \end{array}\right)-\int_{t-h}^{t}{\dot{x}}^{T}(s)R_{1}\dot{x}(s)\;ds\\ & \;+\tau {\dot{x}}^{T}(t)R_{2}\dot{x}(t)-\int_{t-\tau }^{t}{\dot{x}}^{T}(s)R_{2}\dot{x}(s)\;ds\\ & \;+r{\dot{x}}^{T}(t)R_{3}\dot{x}(t)-\int_{t-r}^{t}{\dot{x}}^{T}(s)R_{3}\dot{x}(s)\;ds, \end{array}$$

where

$${\dot{\eta }}_{1}(t)={\left[\begin{array}{ccccc} {\dot{x}}^{T}(t) & {\dot{x}}^{T}(t-\tau ) & x^{T}(t)-x^{T}(t-h) & x^{T}(t)-x^{T}(t-\tau ) & x^{T}(t)-x^{T}(t-r) \end{array}\right]}^{T}.$$

By noting ${\eta_{1}}(t)={\varGamma_{1}}\xi(t)$, ${{\dot{\eta}}_{1}}(t)={\varGamma_{2}}\xi(t)$ and $\dot{x}(t)={e_{0}}\xi(t)$ and by utilizing (4), we obtain

11 $$ - \int_{t - r}^{t} {{x^{T}}(s){Q_{3}}x(s)} \,ds \le \xi^{T} (t) \bigl(- re_{12}^{T}{Q_{3}} {e_{12}}- 3r\varOmega_{1}^{T}{Q_{3}} { \varOmega_{1}} - 5r\varOmega _{2}^{T}{Q_{3}} {\varOmega_{2}}\bigr)\xi(t). $$

Employing Lemma 2.2 to (10), it is not difficult to get

12 $$\begin{aligned}& - \int_{t - h}^{t} {{{\dot{x}}^{T}}(s){R_{1}} \dot{x}(s)} \,ds \le\xi^{T} (t){\varOmega_{3}}\xi(t), \end{aligned}$$

13 $$\begin{aligned}& - \int_{t - \tau}^{t} {{{\dot{x}}^{T}}(s){R_{2}} \dot{x}(s)} \,ds \le\xi^{T} (t){\varOmega_{4}}\xi(t), \end{aligned}$$

and

14 $$ - \int_{t - r}^{t} {{{\dot{x}}^{T}}(s){R_{3}} \dot{x}(s)} \,ds \le\xi^{T} (t){\varOmega_{5}}\xi(t). $$

From the condition ${F^{T}}(t)F(t) \le I $, clearly, we have

15 $$ {p^{T}}(t)p(t) \le{q^{T}}(t)q(t)={ \xi^{T}}(t){\varSigma^{T}}\varSigma\xi(t). $$

Accordingly, there exists a matrix $H>0$, such that the following inequality holds:

16 $$ 0 \le{\xi^{T}}(t){\varSigma^{T}}H\varSigma \xi(t) - {p^{T}}(t)Hp(t), $$

where *Σ* is defined in (7).

From (10) to (16), one can obtain

$$ \dot{V}(t)\le\xi^{T} (t) \varPsi\xi(t). $$

By using the Schur complement, according to the condition (7), it is easy to see that the inequality $\varPsi<0$. Hence, by Theorem 1.3 in [19], system (1) is robustly asymptotically stable. This completes our proof. □

### Remark 3.2

It is worthy to note that the construction on Lyapunov functionals in (8) is very creative. Firstly, the functional contains three single integrals and four double integrals based on the property of the neutral distributed delay systems. Secondly, functionals consider the cross influence between the state information $x(t-\tau)$, and its derivatives and the system. Thirdly, the constructed functionals introduce additional state information because of the utilization of the inequalities in the above lemmas, such as $\frac{1}{{{h^{2}}}}\int_{t - h}^{t} {\int_{\theta}^{t} {{x^{T}} ( s )} } \,ds\,d\theta$, $\frac {1}{{{\tau^{2}}}}\int_{t - \tau}^{t} {\int_{\theta}^{t} {{x^{T}} ( s )} } \,ds\,d\theta$, $\frac{1}{{{h^{3}}}}\int_{t - h}^{t} {\int_{\theta}^{t} {\int _{u}^{t} {{x^{T}} ( s )} } } \,ds\,du\,d\theta$, $\frac{1}{{{\tau ^{3}}}}\int_{t - \tau}^{t} {\int_{\theta}^{t} {\int_{u}^{t} {{x^{T}} ( s )} } } \,ds\,du\,d\theta$, $\frac{1}{{{r^{3}}}}\int_{t - r}^{t} {\int_{\theta}^{t} {\int_{u}^{t} {{x^{T}} ( s )} } } \,ds\,du\,d\theta$ and so on. Since the information of the state can be fully utilized, the resulting stability criteria may be less conservative.

### Remark 3.3

In many practical systems, finding the MAUB on *r* or *h* for different *h* or *r* to analyze the stability of the distributed delay systems is also meaningful.

By utilizing a method like in Theorem 3.1, one obtains the less conservative DDS criterion for the following system with $h\neq r$:

17 $$ \textstyle\begin{cases} \dot{x} ( t ) = Ax ( t ) + {A_{1}}x ( {t - h} ) + {A_{2}}\int_{t - r}^{t} {x ( s )} \,ds , \\ x ( t ) = \phi ( t ),\quad \forall t \in [ { - \sigma,0} ],\sigma=\max \{ {h,r} \},t \ge0. \end{cases} $$

### Corollary 3.4

*For given scalars* *h* *and* *r*, *system* (17) *is asymptotically stable*, *if there exist positive definite matrices* $P\in\mathbb {R}^{3n\times3n}$, $Q_{i}\in\mathbb{R}^{n\times n}$ ($i\in{1,2,3}$), *and* $R_{1}, R_{2}\in\mathbb{R}^{n\times n}$, *and any matrices* $N_{k},M_{k}\in\mathbb{R}^{3n\times n}$ ($k\in{1,2}$) *satisfying the following inequalities*:

18 $$\begin{aligned} \bar{\varPsi}&= \operatorname{sym} \bigl\{ {\varGamma_{4}^{T}P{ \varGamma_{3}}} \bigr\} + e_{1}^{T} ( {{Q_{1}} + {Q_{2}} + r{Q_{3}}} ){e_{1}}- e_{2}^{T}{Q_{1}} {e_{2}} - e_{3}^{T}{Q_{2}} {e_{3}} \\ &\quad {}- re_{5}^{T}{Q_{3}} {e_{5}}+ he_{0}^{T}{R_{1}} {e_{0}}+ re_{0}^{T}{R_{2}} {e_{0}}+ { \varOmega_{6}} +{\varOmega_{7}}< 0, \end{aligned}$$

*where*

$$\begin{array}{rl} & \Gamma_{3}={\left[\begin{array}{ccc} e_{1}^{T} & he_{4}^{T} & re_{5}^{T} \end{array}\right]}^{T},\;\Gamma_{4}={\left[\begin{array}{ccc} e_{0}^{T} & e_{1}^{T}-e_{2}^{T} & e_{1}^{T}-e_{3}^{T} \end{array}\right]}^{T},\\ & e_{0}=Ae_{1}+A_{1}e_{2}+rA_{2}e_{5},\;{\bar{\upsilon }}_{1}={\left[\begin{array}{ccc} e_{1}^{T} & e_{2}^{T} & e_{4}^{T} \end{array}\right]}^{T},\;{\bar{\upsilon }}_{2}={\left[\begin{array}{ccc} e_{1}^{T} & e_{3}^{T} & e_{5}^{T} \end{array}\right]}^{T},\\ & \Omega_{6}={\bar{\upsilon }}_{1}^{T}\left(\sum_{k=1}^{2}\frac{h}{2k-1}N_{k}R_{1}^{-1}N_{k}^{T}\right){\bar{\upsilon }}_{1}+\mathrm{sym}\left\{{\bar{\upsilon }}_{1}^{T}N_{k}E_{k}\right\},\\ & \Omega_{7}={\bar{\upsilon }}_{2}^{T}\left(\sum_{k=1}^{2}\frac{r}{2k-1}M_{k}R_{2}^{-1}M_{k}^{T}\right){\bar{\upsilon }}_{2}+\mathrm{sym}\left\{{\bar{\upsilon }}_{2}^{T}M_{k}D_{k}\right\},\\ & E_{1}=e_{1}-e_{2},\;E_{2}=e_{1}+e_{2}-2e_{4},\\ & D_{1}=e_{1}-e_{3},\;D_{2}=e_{1}+e_{3}-2e_{5},\\ & e_{i}=\left[\begin{array}{ccc} 0_{n\times (i-1)n} & I_{n} & 0_{n\times (5-i)n} \end{array}\right],\;i=1,2,3,4,5. \end{array}$$

### Proof

Choose LKF as follows:

$$\begin{aligned} \tilde{V}(t) & = \eta_{2}^{T}(t)P{\eta_{2}}(t) + \int_{t - h}^{t} {{x^{T}}(s){Q_{1}}x(s)} \,ds + \int_{t - r}^{t} {{x^{T}}(s){Q_{2}}x(s)} \,ds \\ &\quad {} + \int_{t - r}^{t} { \int_{\theta}^{t} {{x^{T}} ( s ){Q_{3}}x ( s )\,ds\,d\theta} } + \int_{t - h}^{t} { \int_{\theta}^{t} {{{\dot{x}}^{T}}(s){R_{1}} \dot{x}(s)} \,ds} \,d\theta \\ &\quad {} + \int_{t - r}^{t} { \int_{\theta}^{t} {{{\dot{x}}^{T}}(s){R_{2}} \dot{x}(s)} \,ds} \,d\theta, \end{aligned}$$

where

$$\begin{array}{c} \eta_{2}(t)={\left[\begin{array}{ccc} x^{T}(t) & \int_{t-h}^{t}x^{T}(s)\;ds & \int_{t-r}^{t}x^{T}(s)\;ds \end{array}\right]}^{T},\\ \xi_{1}(t)={\left[\begin{array}{ccccc} x^{T}(t) & x^{T}(t-h) & x^{T}(t-r) & \frac{1}{h}\int_{t-h}^{t}x^{T}(s)\;ds & \frac{1}{r}\int_{t-r}^{t}x^{T}(s)\;ds \end{array}\right]}^{T}. \end{array}$$

Combining (3) with (5), and following a similar line to Theorem 3.1, it is not hard to obtain the DDS criterion (18). The proof is completed. □

In the case of $r=h$, system (1) is rewritten as

19 $$ \dot{x} ( t ) - B\dot{x} ( {t - \tau} ) = Ax ( t ) + {A_{1}}x ( {t - h} )+ {A_{2}} \int_{t - h}^{t} {x ( s )} \,ds. $$

### Theorem 3.5

*For given scalars* *τ* *and* *h*, *system* (19) *is asymptotically stable*, *if there exist matrices* $P\in\mathbb{R}^{5n\times5n}$, $Q_{1}, Q_{2}, R\in\mathbb{R}^{n\times n}$, *and any matrices* $M_{k}\in\mathbb{R}^{6n\times n}$ ($k\in{1,2,3,4,5}$) *satisfying the following inequalities*:

20 $$\begin{aligned} \varPhi&= \operatorname{sym} \bigl\{ {\varUpsilon_{2}^{T}P{ \varUpsilon_{1}}} \bigr\} + e_{1}^{T}{Q_{1}} {e_{1}} - e_{2}^{T}{Q_{1}} {e_{2}} + e_{0}^{T}{Q_{2}} {e_{0}}- e_{7}^{T}{Q_{2}} {e_{7}} \\ &\quad {}+ he_{0}^{T}R{e_{0}} +{ \upsilon^{T}}\varOmega\upsilon< 0, \end{aligned}$$

*where*

$$\begin{array}{rl} & {ϒ}_{1}={\left[\begin{array}{ccccc} e_{1}^{T} & he_{3}^{T} & \frac{h^{2}}{2}e_{4}^{T} & \frac{h^{3}}{6}e_{5}^{T} & \frac{h^{4}}{24}e_{6}^{T} \end{array}\right]}^{T},\\ & {ϒ}_{2}={\left[\begin{array}{ccccc} e_{0}^{T} & e_{1}^{T}-e_{2}^{T} & h(e_{1}^{T}-e_{3}^{T}) & \frac{h^{2}}{2}(e_{1}^{T}-e_{4}^{T}) & \frac{h^{3}}{6}(e_{1}^{T}-e_{5}^{T}) \end{array}\right]}^{T},\\ & e_{0}=Ae_{1}+A_{1}e_{2}+hA_{2}e_{3}+Be_{7},\\ & e_{i}=\left[\begin{array}{ccc} 0_{n\times (i-1)n} & I_{n} & 0_{n\times (7-i)n} \end{array}\right],\;i=1,2,\ldots ,7. \end{array}$$

*υ* *and* *Ω* *are defined in Lemma *2.2.

### Proof

Consider the LKF candidate

21 $$\begin{aligned} {\bar{V}}(t)&=\eta_{3}^{T}(t)P{\eta_{3}}(t) + \int_{t - h}^{t} {{x^{T}}(s){Q_{1}}x(s)} \,ds + \int_{t - \tau}^{t} {{{\dot{x}}^{T}}(s){Q_{2}} \dot{x}(s)} \,ds \\ &\quad {}+ \int_{t - h}^{t} { \int_{\theta}^{t} {{{\dot{x}}^{T}}(s)R\dot{x}(s)} \,ds} \,d\theta, \end{aligned}$$

where

$$\begin{array}{rl} & \eta_{3}(t)={\left[\begin{array}{ccccc} x^{T}(t) & v_{1}^{T}(t) & v_{2}^{T}(t) & v_{3}^{T}(t) & v_{4}^{T}(t) \end{array}\right]}^{T},\;v_{1}(t)=\int_{t-h}^{t}x(s)\;ds,\\ & v_{2}(t)=\int_{t-h}^{t}\int_{\theta }^{t}x(s)\;ds\;d\theta ,\;v_{3}(t)=\int_{t-h}^{t}\int_{\theta }^{t}\int_{u}^{t}x^{T}(s)\;ds\;du\;d\theta ,\\ & v_{4}(t)=\int_{t-h}^{t}\int_{\theta }^{t}\int_{u}^{t}\int_{v}^{t}x^{T}(s)\;ds\;dv\;du\;d\theta . \end{array}$$

Define

$$\xi_{2}(t)={\left[\begin{array}{ccccccc} x^{T}(t) & x^{T}(t-h) & \frac{1}{h}v_{1}^{T}(t) & \frac{2}{h^{2}}v_{2}^{T}(t) & \frac{6}{h^{3}}v_{3}^{T}(t) & \frac{24}{h^{4}}v_{4}^{T}(t) & {\dot{x}}^{T}(t-\tau ) \end{array}\right]}^{T}.$$

By noting ${\eta_{3}}(t)={\varUpsilon_{1}}{\xi_{2}}(t)$, $\dot{\eta}_{3}^{T}(t)={\varUpsilon_{2}}{\xi_{2}}(t)$ and $\dot{x}(t)={e_{0}}{\xi_{2}}(t)$, the derivative of ${\bar{V}}(t)$ along the trajectory of system (19) is computed:

22 $$\begin{aligned} \dot{\bar{V}}(t)&= 2\dot{\eta}_{3}^{T}(t)P{ \eta_{3}}(t) + {x^{T}}(t){Q_{1}}x(t) - {x^{T}}(t - h){Q_{1}}x(t - h) + {{\dot{x}}^{T}}(t){Q_{2}} \dot{x}(t) \\ &\quad {} - {{\dot{x}}^{T}}(t - \tau){Q_{2}}\dot{x}(t - \tau)+ h{{\dot{x}}^{T}}(t)R\dot{x}(t)- \int_{t - h}^{t} {{{\dot{x}}^{T}}(s)R\dot{x}(s)} \,ds. \end{aligned}$$

Using Lemma 2.2, one can see that the following inequality holds:

23 $$ - \int_{t - h}^{t} {{{\dot{x}}^{T}}(s)R\dot{x}(s)} \,ds \le\xi_{2}^{T}(t) \Biggl[ \sum _{k = 1}^{5} {\frac{h}{{2k - 1}}{M_{k}} {R^{ - 1}}M_{k}^{T}}+ \sum _{k = 1}^{5} {\operatorname{sym} \{ {{M_{k}} {\varPi_{k}}} \}} \Biggr]{\xi_{2}}(t). $$

Therefore, by combining (22) and (23), one has

$$ \dot{\bar{V}}(t)\le\xi_{2}^{T}(t)\varPhi{ \xi_{2}}(t). $$

We proceed by applying the Schur complement, and *Φ* is given in Theorem 3.5. Thus, if $\varPhi<0$ in (20) holds, it is easy to see that $\dot{\bar{V}}(t)<0$, which means that system (19) has asymptotic stability. This completes the proof. □

### Remark 3.6

Unlike the previous conditions, the quadruple integral information of the state has been fully utilized in Theorem 3.5. In order to coordinate the application of the inequality in Lemma 2.2, both the quadratic vector ${\eta_{3}}(t)$ and the intermediate vector ${\xi _{2}}(t)$ are expanded by adding the quadruple integral term ${v_{4}}(t)$. What is more important is the fact that adding the quadruple integral term can make the result less conservative in Theorem 3.5. As a matter of fact, the novel constructed Lyapunov–Krasovskii functionals are as important to get less conservative delay-dependent stability conditions as skillfully using more accurate integral inequalities.

For system (19), if there exist uncertain matrices Δ*A*, $\Delta{A_{1}}$, $\Delta{A_{2}}$ and Δ*B*, we may also obtain the sufficient condition to ensure the robustly asymptotically stability. Hence, we will consider the uncertain neutral delayed system as follows:

24 $$ \dot{x}(t) - \mathbf{B}\dot{x}(t - \tau) = \mathbf{A}x(t) + { \mathbf{A}_{1}}x(t - h)+ {\mathbf{A}_{2}} \int_{t - h}^{t} {x(s)} \,ds. $$

From (24), by using similar techniques to Theorem 3.1 to handle the uncertainties, a robust asymptotic stability condition can be given as follows.

### Corollary 3.7

*For given scalars* *τ* *and* *h*, *the uncertain system* (24) *with* ${A_{1}} + \Delta{A_{1}}=0$ *is robustly asymptotically stable*, *if there exist matrices* $P\in\mathbb{R}^{5n\times5n}$, $Q_{1}, Q_{2}, R, K\in\mathbb{R}^{n\times n}$, *and any matrices* $M_{k}\in\mathbb {R}^{6n\times n}$ ($k\in{1,2,3,4,5}$) *satisfying the following inequalities*:

25 $$ \bar{\varPhi}=\varPhi+{\bar{\varSigma}^{T}}K{\bar{\varSigma}} - e_{8}^{T}K{e_{8}}< 0, $$

*where* *Φ* *is defined in* (20) *and*

$$\begin{array}{c} e_{0}=Ae_{1}+hA_{2}e_{3}+Be_{7}+De_{8},\;\bar{\Sigma }=N_{a}e_{1}+hN_{c}e_{3}+N_{d}e_{7},\\ e_{i}=\left[\begin{array}{ccc} 0_{n\times (i-1)n} & I_{n} & 0_{n\times (8-i)n} \end{array}\right],\;i=1,2,\ldots ,8, \end{array}$$

*where*

$$\xi_{3}(t)=\left[\begin{array}{cccccccc} x^{T}(t) & x^{T}(t-h) & \frac{1}{h}v_{1}^{T}(t) & \frac{2}{h^{2}}v_{2}^{T}(t) & \frac{6}{h^{3}}v_{3}^{T}(t) & \frac{24}{h^{4}}v_{4}^{T}(t) & {\dot{x}}^{T}(t-\tau ) & p^{T}(t) \end{array}\right].$$

Similar to the proof of Theorem 3.1, Corollary 3.7 can be proved, so it is omitted here.

### Remark 3.8

This paper just discusses the delay-dependent stability criteria, in which some useful approach can be effectively used to analyze the stabilization for linear neutral systems with time-delay. Moreover, the employed results and methods may be extended to many interesting dynamical models such as uncertain neutral systems with Markovian jumping parameters and uncertain neutral systems with nonlinear perturbations or time-varying delays.

### Remark 3.9

Although the asymptotic stability of system (1) is discussed in this paper, the exponential stability of system (1) can easily be obtained by using a similar proof in [53]. In fact, for a linear neutral system with time-delay, the asymptotic stability implies exponential stability.

## Numerical examples

In this section, several examples are provided to illustrate the usefulness of the above results.

### Example 4.1

Consider system (1) with the following parameters [32]:

$$\begin{array}{rl} & A=\left(\begin{array}{cc} -0.9 & 0.2\\ 0.1 & -0.9 \end{array}\right),\;A_{1}=\left(\begin{array}{cc} -1.1 & -0.2\\ -0.1 & -1.1 \end{array}\right),\\ & A_{2}=\left(\begin{array}{cc} -0.12 & -0.12\\ -0.12 & 0.12 \end{array}\right),\;B=\left(\begin{array}{cc} -0.2 & 0\\ 0.2 & -0.1 \end{array}\right),\\ & D=I,\;N_{a}=N_{b}=N_{c}=N_{d}=0.1I. \end{array}$$

For this example, suppose $\tau=0.1$, by applying Theorem 3.1, one can obtain the MAUB on *r* and *h* for different *h* and *r*, which is enumerated in Table 1 and Table 2, respectively.

**Table 1:** The MAUB of *r* for $\tau=0.1$ and different values *h* in Example 4.1

*h*	0.1	0.5	1.0	1.5	1.6	1.7
[32]	6.64	5.55	1.62	–	–	–
[33]	6.67	6.12	2.75	1.31	0.93	0.42
[34]	6.65	6.02	2.68	0.88	–	–
[35]	6.67	5.83	2.97	1.53	1.33	1.14
[41]	6.67	6.67	5.52	1.95	1.55	1.46
Theorem 3.1	8.64	8.64	6.69	2.66	2.32	2.04

**Table 2:** The MAUB of *h* for $\tau=0.1$ and different values *r* in Example 4.1

*r*	1	2	3	4	5	6
[32]	1.12	0.93	0.77	0.65	0.55	0.43
[33]	1.58	1.20	0.95	0.77	0.64	0.51
[34]	1.47	1.14	0.95	0.80	0.68	0.50
[35]	1.78	1.30	0.99	0.77	0.62	0.47
[41]	1.90	1.49	1.34	1.20	1.07	0.91
Theorem 3.1	2.10	1.71	1.41	1.25	1.14	1.05

### Example 4.2

Consider system (17) with the following parameters [41]:

$$A=\left(\begin{array}{cc} -0.9 & 0\\ 0 & -0.9 \end{array}\right),\;A_{1}=\left(\begin{array}{cc} -1 & -0.12\\ 0.12 & -1 \end{array}\right),\;A_{2}=\left(\begin{array}{cc} -0.12 & -0.12\\ -0.12 & 0.12 \end{array}\right).$$

For this example, in the case $h=1$, the allowable delay bounds on *r* in [41] is computed as 11.05, but by Corollary 3.4, we can get a larger delay bound, 11.21. Besides, in the case $r=1$, the allowable delay bounds on *h* in [36, 37] and [41] are computed as 1.8302, 2.8011 and 3.5823, respectively. However, by Corollary 3.4, one can obtain a larger delay bound 3.9235, which are 114.375%, 40.070%, 9.524% more than that of [36, 37] and [41], respectively. In addition, this example shows that our method in this paper is better than that in [36, 37] and [41].

### Example 4.3

Consider system (19) with the following parameters when $B=0$:

$$A=\left(\begin{array}{cc} -2 & 0\\ 0 & -0.9 \end{array}\right),\;A_{1}=\left(\begin{array}{cc} -1 & 0\\ -1 & -1 \end{array}\right),\;A_{2}=0.$$

Both the conservatism and the computation burden are carefully compared among different stability conditions. The MAUB is listed in Table 3. It is easy to see that the maximum admissible upper bounds obtained by Theorem 3.5 is the largest. It is worth to mention that, by Theorem 3.5, the obtained MAUB is the analytical value. This clearly shows the effectiveness of the DDS criteria in Theorem 3.5.

**Table 3:** Upper bound on *h* obtained for Example 4.3

Methods	$h_{\mathrm{max}}$
(*N* = 1) [19]	6.059
(*N* = 2) [19]	6.165
[47]	6.1107
[43]	6.059
(*N* = 6) [48]	6.12
[49]	6.1664
[46]	6.1719
Theorem 3.5	6.1725
The analytical bounds	6.1725

### Example 4.4

Consider system (19) with the following parameters when $B=0$:

$$A=\left(\begin{array}{cc} 0 & 1\\ -2 & 0.1 \end{array}\right),\;A_{1}=\left(\begin{array}{cc} 0 & 0\\ 1 & 0 \end{array}\right),\;A_{2}=\left(\begin{array}{cc} 0 & 0\\ 0 & 0 \end{array}\right).$$

Since $\operatorname{Re} ( {eig(A + {A_{1}})} ) = 0.05>0$, the system is unstable when $h=0$. Few linear matrix inequalities can test the stability condition of this system. This is so because the delay is distributed in some interval to guarantee the stability for the systems. Table 4 lists the results obtained from Theorem 3.5 and other conditions reported in the literature. From Table 4, we can see that our maximum admissible upper bounds are the analytical bounds.

**Table 4:** Delay interval which stability of system in Example 4.4 is guaranteed

Methods	$h_{\mathrm{min}}$	$h_{\mathrm{max}}$
(*N* = 1) [19]	0.1006	1.4272
(*N* = 2) [19]	0.1003	1.6921
[43]	0.1003	1.5406
[50]	0.1002	1.5954
[49]	0.100169	1.7122
[46]	0.100169	1.7177
Theorem 3.5	0.100169	1.7178
The analytical bounds	0.100169	1.7178

### Example 4.5

When the parameters of system (19) in this example is described as follows ($B=0$):

$$A=\left(\begin{array}{cc} 0.2 & 0\\ 0.2 & 0.1 \end{array}\right),\;A_{1}=0,\;A_{2}=\left(\begin{array}{cc} -1 & 0\\ -1 & -1 \end{array}\right).$$

The corresponding delay bounds can also be given by computing the linear matrix inequality in Theorem 3.5. From Table 5, in order to gain the MAUB to guarantee the system stability, various kinds of methods are proposed to calculate the MAUB. It is easily seen that our presented approach is best due to the fact that the obtained MAUB concerns the analytical bounds.

**Table 5:** Upper bound on *h* obtained for Example 4.5

Methods	$h_{\mathrm{max}}$
[37]	1.6339
[43]	1.8770
[50]	1.9504
[51]	2.0395
[49]	2.0395
[52]	2.0402
[46]	2.0412
Theorem 3.5	2.0412
The analytical bounds	2.0412

### Example 4.6

To show the comparison in more detail, the parameters of the system (19) in this example are given by [43] when $B=0$:

$$A=\left(\begin{array}{cc} 0 & 1\\ -100 & -1 \end{array}\right),\;A_{1}=\left(\begin{array}{cc} 0 & 0.1\\ 0.1 & 0.2 \end{array}\right),\;A_{2}=0.$$

By using Theorem 3.5, a larger delay bound 0.7495 is got. In order to show more clearly, Table 6 lists the computed upper bounds by unlike methods. It is easily observed that our method produces better result than the existing results.

**Table 6:** Upper bound on *h* obtained for Example 4.6

Methods	$h_{\mathrm{max}}$
[50]	0.126
[51]	0.577
[52]	0.675
Theorem 3.5	0.749

### Example 4.7

Consider system (19) with the following parameters:

$$A=\left(\begin{array}{cc} -2 & 0\\ 0 & -0.9 \end{array}\right),\;A_{1}=0,\;A_{2}=\left(\begin{array}{cc} -1 & 0\\ -1 & -1 \end{array}\right),\;B=\left(\begin{array}{cc} -0.2 & 0\\ 0.2 & -0.1 \end{array}\right).$$

By Corollary 1 in [37], the system (19) is asymptotically stable for all delays $h \in [ {0,1.8536} ]$. Applying Theorem 3.5 to this example, system (19) becomes asymptotically stable for all delays $h \in [ {0,3.6574} ]$.

### Example 4.8

Consider system (24) with the following parameters:

$$\begin{array}{rl} & A=\left(\begin{array}{cc} -2 & 0\\ 0 & -0.9 \end{array}\right),\;A_{2}=\left(\begin{array}{cc} -1 & 0\\ -1 & -1 \end{array}\right),\;B=\left(\begin{array}{cc} -0.2 & 0\\ 0.2 & -0.1 \end{array}\right),\\ & D=I,\;N_{a}=N_{c}=0.1I. \end{array}$$

In the first place, we take ${N_{d}}=0$. By Corollary 1 in [37], the system (24) is robustly asymptotically stable for all delays $h \in [ {0,1.7174} ]$. However, using Corollary 3.7 in this example, the interval of delay *h* such that the system (24) is robustly asymptotically stable is $[ {0,3.2314} ]$. Then let ${N_{d}}=0.1I$, by Corollary 2 in [37], the system (24) is robustly asymptotically stable for $h \in [ {0,1.4986} ]$. However, by applying Corollary 3.7, we can obtain the interval of delay $h \in [ {0,3.0175} ]$ which ensures the robust stability of system (24).

### Example 4.9

To show the comparison in more detail, the parameters of the system (19) in this example is given by [18]

$$A=\left(\begin{array}{cc} -3 & -2\\ 1 & 0 \end{array}\right),\;A_{1}=\left(\begin{array}{cc} 0 & \alpha \\ \alpha & 0 \end{array}\right),\;A_{2}=0,\;B=\left(\begin{array}{cc} 0.1 & 0\\ 0 & 0.1 \end{array}\right),$$

where *α* is a real parameter. In this example, we assume $\tau =h=1$. By Theorems 2.5 and 2.6 in [18], it can be found that this system (19) is not only asymptotic but exponential for all $|\alpha|\le0.6213$. However, by Theorem 3.5, it is computed that the maximum allowed value $|\alpha|\le1$. Therefore, Theorem 3.5 is less conservative than the results in [18].

## Conclusion

Based on some new LKF and IIs, several new stability and RSCSs have been proposed for uncertain linear neutral system with time-delay. The obtained DDS conditions in this paper are of low conservatism owing to the constructed novel LKF, which combines with the new II technique. Besides, the problem of robust stability for uncertain systems without neutral or distributed term are commendably addressed. Moreover, some interesting examples have been presented to display the low conservatism of the derived DDS conditions by comparison with the existing results.
